# Sudor Cruentus or Blood Sweating

**Published:** 1882-01

**Authors:** John N. Navin


### II.—SUDOR CRUENTUS, OR BLOOD SWEATING

REPORTED BY JOHN N. NAVIN, V.S.

The strange horse disease which I mentioned in my letter to you is a most singular one, at least to me; one which I never read of or met with. The one I saw, a four-year-old, half-breed Clydesdale colt, untrained. The only visible symptoms were an inclination to keep lying down constantly and frequency of his pulse, which marked 70. Eat hay while kept up, but on being left alone, resumed his favorite attitude; by turning round much in the fashion of a dog, evidently stiff, but after once or twice turning round, dropped down and continued so until urged up. The owner remarked that he had not yet commenced to sweat blood, and on expressing my surprise at the revelation, both the owner and his man assured me that those horses which died, one, a mare on the farm, sweated blood which, when scraped up on to a plate with a knife, appeared like water mixed with one-third the quantity of blood; that for three or four days, while the mare lived, it became darker and thicker, I mean more blood and less serum, until death, which, if I remember correctly, was four days, the duration of the disease being about seven days. The owner remarked to me that the colt had not yet arrived at the period at which all the others had commenced to sweat—first a watery fluid, which soon changed to a reddish color—and that they seldom stretch out in the first stage of the disease.

Before being told of the bloody effusion I inquired if a post-mortem examination revealed any strange character in any of the internal organs. The owner said that the heart of the mare contained a mass of yellowish, jelly-like mass. On stating my opinion that she had died of clot in the heart, which, when it broke from its walls, caused death by being forced into the lungs, he told me that he cut the wall of the aorta and other arteries off from their connection with the heart, and found that what they contained resembled a tallow candle more than any other thing, but more yellow in color. It lengthened and contracted like India rubber or a spiral spring, and would stretch one-fourth its length, when pulled, before breaking.

---

On expressing my surprise at what became of the red portion of the blood, he then related the passage of it through the pores of the skin, and other farmers who lost stock by the same disease related the same symptoms. I gave tincture of aconite, spts. vini rect. and tr. belladonnæ to lower the pulse, and iron, hoping to correct the division of the constituents of the blood—in fact, I misunderstood the nature of the disease entirely.

I told the owner of the horse to write me daily; that, if necessary, I

would visit the horse, and that should he dieI would not charge for investigating the case, but I never since heard from him.

INDIANAPOLIS, Ind.

---

A disease has been met with in human beings of rare occurrence, under the name of Sudor Anglicus, which, when very severe, is accompanied by hemorrhagic perspiration. This disease first appeared in England in 1486, and recurred at intervals until the middle of the sixteenth century. This form is said to have terminated either in death or recovery in twenty-four hours. The disease again recurred in 1821, and was described at length by M. Rayner. When the perspiration was dark in color or blood-stained, it was called Sudor Anglicus Niger, or black English sweating sickness. It seems, better, however, to apply the name "sudor cruentus," or sweating of blood, or bloody sweating, hemorrhage from the skin. Both the French and Germans have observed this disease, but it seems to have been limited to small geographical territories. As the intervals between epidemics have been long, many writers have never met with a case, and therefore infer that it never occurs.

The anatomical lesions in the human subject seem to be a little different from those hinted at by Dr. Navin. It is a great pity that necropsies could not have been made in these cases, and the gross findings worked up microscopically, for it is from comparative pathology that great advances in medicine must spring.—[ED.

